# Pulmonary arteriovenous malformations in Rendu-Osler-Weber syndrome

**DOI:** 10.1590/1677-5449.202301332

**Published:** 2024-04-08

**Authors:** Cristiane Ferreira de Araújo-Gomes, Carlos Eduardo Virgini-Magalhães, Leonardo Silveira de Castro, Eduardo de Oliveira Rodrigues, Alex Antunes Bezerra, Monica Rochedo Mayall, Cristina Ribeiro Riguetti-Pinto, Felipe Borges Fagundes

**Affiliations:** 1 Universidade do Estado do Rio de Janeiro - UERJ, Hospital Universitário Pedro Ernesto - HUPE, Rio de Janeiro, RJ, Brasil.

**Keywords:** embolization, therapeutic, arteriovenous fistula, telangiectasia, hereditary hemorrhagic, Rendu-Osler-Weber

## Abstract

Rendu-Osler-Weber syndrome, also known as hereditary hemorrhagic telangiectasia, is an autosomal dominant hereditary disorder. It is characterized by presence of multiple arteriovenous malformations (AVMs) and telangiectasias. This article reports two cases of patients with Rendu-Osler-Weber syndrome who had pulmonary AVMs and underwent successful endovascular treatment. A brief review of the literature shows that up to 50% of patients with the syndrome have pulmonary AVMs and there is usually a positive family history in these patients. These pulmonary AVMs are multiple in 30% of cases and are associated with the most severe disease complications. Most patients are asymptomatic, even in the presence of AVMs with right-left shunts. When these shunts exceed 25% of the total blood volume, dyspnea, cyanosis, digital clubbing, and extracardiac murmurs may occur. Endovascular treatment is safe and offers control of complications from hereditary hemorrhagic telangiectasia and is currently the treatment of choice for these lesions.

## INTRODUCTION

Rendu-Osler-Weber syndrome (ROWS), or hereditary hemorrhagic telangiectasia (HHT), is an autosomal dominant disease with estimated prevalence of 1/5,000.^1^ It is characterized by presence of multiple arteriovenous malformations (AVMs) and telangiectasias. These AVMs may develop or expand over time in the liver, lungs, and brain. They frequently form large left-right (L-R) shunts, increasing cardiac output, causing desaturation of arterial blood, and giving rise to venous emboli that can reach the peripheral circulation.^2^ ROWS shortens life expectancy by an average of 7 years.^3^

This study describes endovascular treatment of two cases of patients with ROWS and pulmonary AVMs and presents a brief review of the literature on this rare disease.

## PART IA - CLINICAL SITUATION

The patient was a 47-year-old female whose clinical presentation began in 2018 with progressive dyspnea and falling oxygen saturation (SaO_2_). Clinical work-up revealed normal pulmonary auscultation, dyspnea in response to medium effort, and digital clubbing. Her prior history of disease included an ischemic stroke at 26 years of age, with no motor or functional sequelae, hospital admission for pneumonia, requiring mechanical ventilation, and prior surgery due to renal and hepatic abscesses, 11 months previously. She also reported a history of prior treatment for erythrocytosis, leukopenia, thrombocytopenia, and polyglobuly.

**Figure 1:**
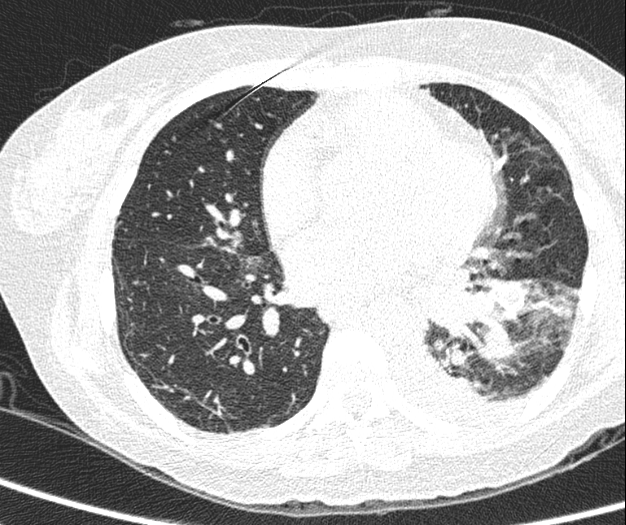
Angiotomography of the chest (case 1) with hypervascularized nodular lesions characteristic of arteriovenous malformations.

A chest angiotomography (angio-CT) confirmed the image of AVMs (Figure 1) with normal spirometry. Her deteriorating respiratory status with dyspnea in response to minimal effort and SaO_2_ less than 65% in room air imposed a dilemma: conservative treatment dependent on home oxygen therapy or intervention? The best of the available intervention options was embolization of the AVMs, since conventional surgical treatment was associated with greater risk and morbidity for the clinical case in question.

## PART IIA - WHAT WAS DONE

The patient was admitted for treatment of pulmonary AVMs. The procedure was performed under general anesthesia, with the patient maintaining SaO_2_ at around 80% throughout the operation, despite oxygen therapy.

**Figure 2:**
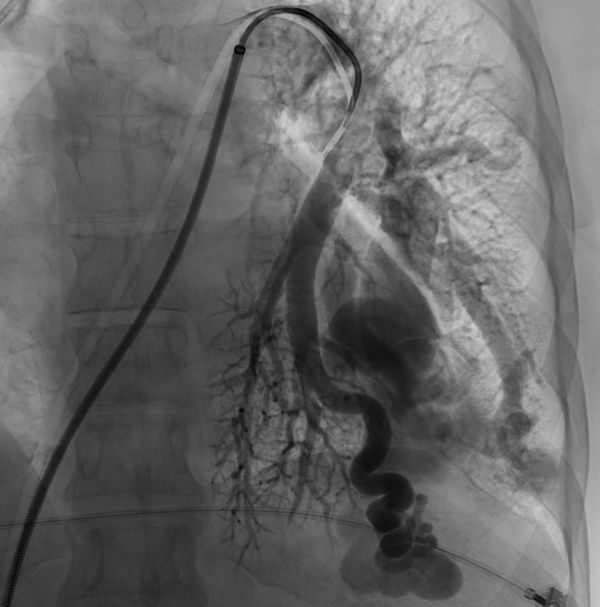
Selective arteriography identifying voluminous pulmonary arteriovenous malformations (case 1).

**Figure 3:**
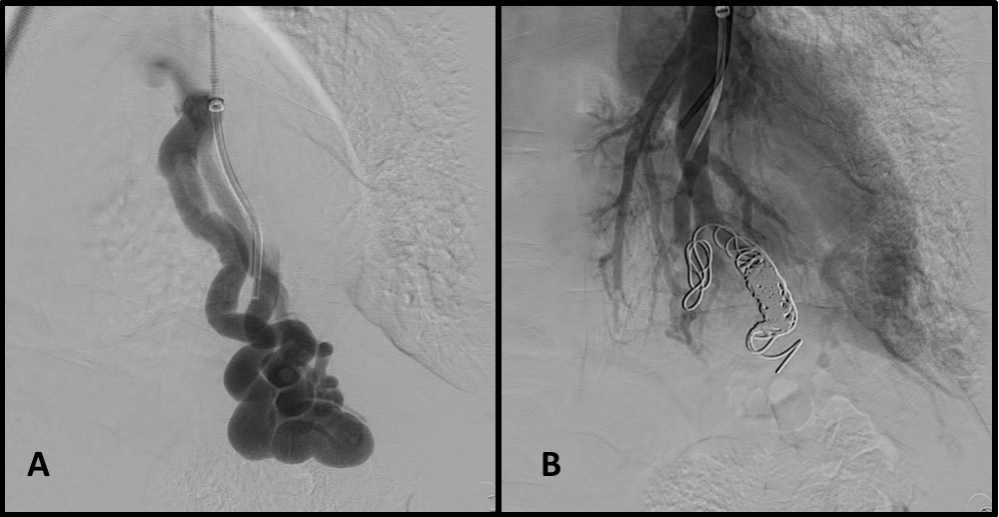
Embolization of arteriovenous malformations in case 1. (A) Selective catheterization of the arteriovenous malformation in the lower right lobe; and (B) result immediately after embolization with six pushable coils.

**Figure 4:**
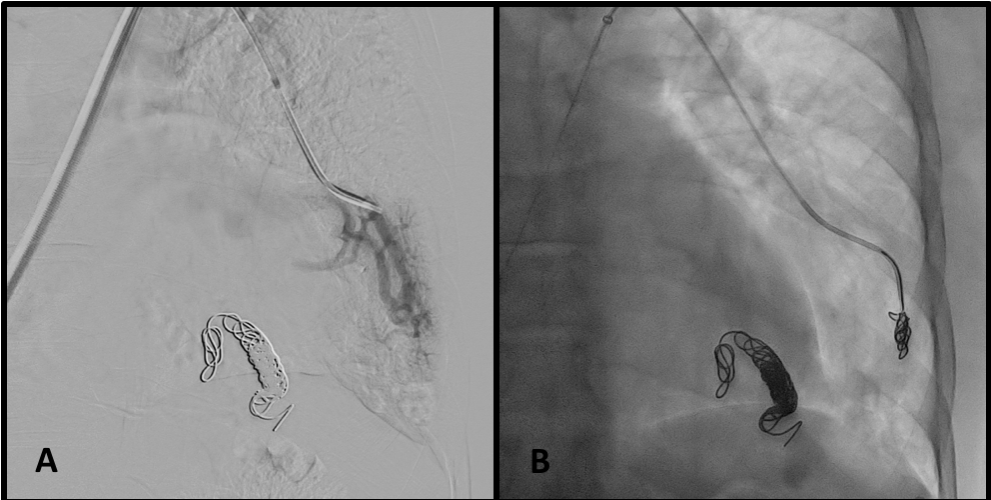
Embolization of arteriovenous malformations in case 1. (A) Selective catheterization of peripheral arteriovenous malformation; and (B) deployment of pushable coil.

We obtained femoral access with the Seldinger technique and catheterized the pulmonary arteries, identifying the AVMs (Figure 2). Each AVM underwent selective catheterization and embolization according to its diameter. The AVM in the lower left lobe was embolized with six Nester pushable coils ^®^ 14-12 (Figure 3A and 3B). The smaller caliber, peripheral AVM was embolized with a single Nester pushable coil ^®^ 14-12 (Figure 4A and 4B).

**Figure 5:**
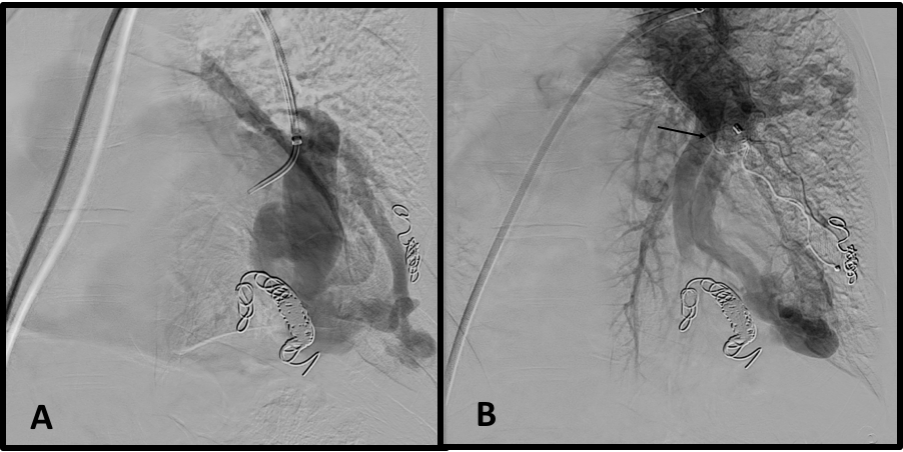
Embolization of arteriovenous malformations in case 1. (A) Selective catheterization of voluminous arteriovenous malformation; and (B) deployment of Amplatzer^®^ plug. Detail (arrow) of deployment of the last ring of the plug to occlude the smaller caliber arteriovenous malformation.

Other large caliber AVMs (Figure 5A) were embolized together with a 16 mm Amplatzer II device. We used the last ring of the plug to occlude the ostium of the artery feeding the smaller AVM (Figure 5B, arrow). SaO2 increased to 96% immediately after release of the devices.

On the second postoperative day (POD), the patient suffered intense chest pain, which was treated with analgesia, achieving full resolution within 48 hours. She was discharged from hospital on the fifth POD, maintaining SaO_2_ at 96%. Four years after the intervention, the patient remains in outpatient follow-up with the pulmonology service, is clinically stable, and has preserved functional capacity.

## PART IB - CLINICAL SITUATION

**Figure 6:**
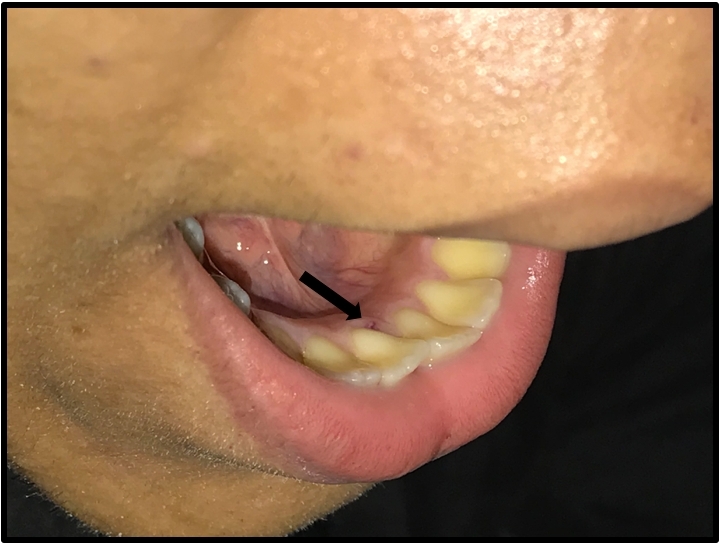
Physical examination of the patient (case 2) - normal, except for a small telangiectasia in the oral cavity.

An asymptomatic 20-year-old male patient was referred for clinical investigation of a radiological finding of a pulmonary mass in the right hemithorax during an admission exam. During the guided patient history, he reported self-limiting nosebleed episodes during childhood and adolescence and bleeding from the gingiva and tongue, without repercussions. He had a positive family history of similar episodes of nosebleeding. The only physical examination finding was a small telangiectasia in the oral cavity (Figure 6).

**Figure 7:**
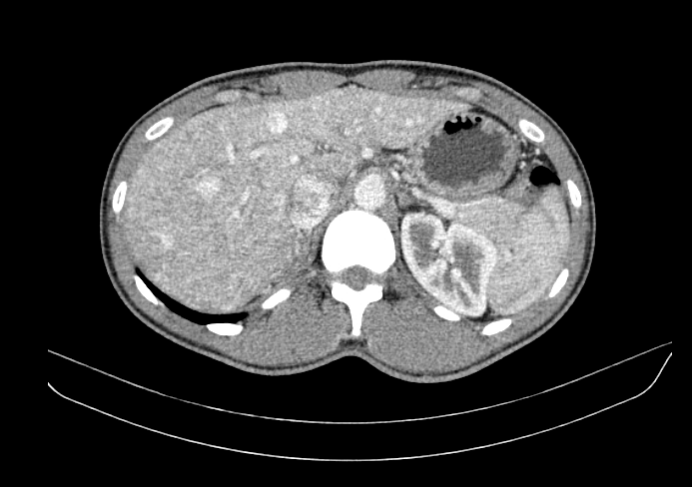
Angiotomography identified multiple nodular hypervascularized images in the hepatic parenchyma compatible with arteriovenous malformations (case 2).

A transthoracic echocardiogram using the microbubbles technique revealed an ejection fraction of 69%, with an L-R pulmonary shunt. An upper digestive endoscopy found evidence of gastric vascular angiectasias and small caliber esophageal varicose veins (BAVENO VI). A chest angio-CT confirmed AVM in the upper and lower lobes of the right lung and also identified multiple hypervascularized nodular images in the liver, also compatible with AVMs (Figure 7).

The dilemma in this case, in an asymptomatic patient, was to decide between conservative management and prophylactic intervention in the lesions. In view of the risk of major bleeding from the lesion, and because this was a young patient, we recommended endovascular treatment of the pulmonary lesions.

## PART IIB - WHAT WAS DONE

**Figure 8:**
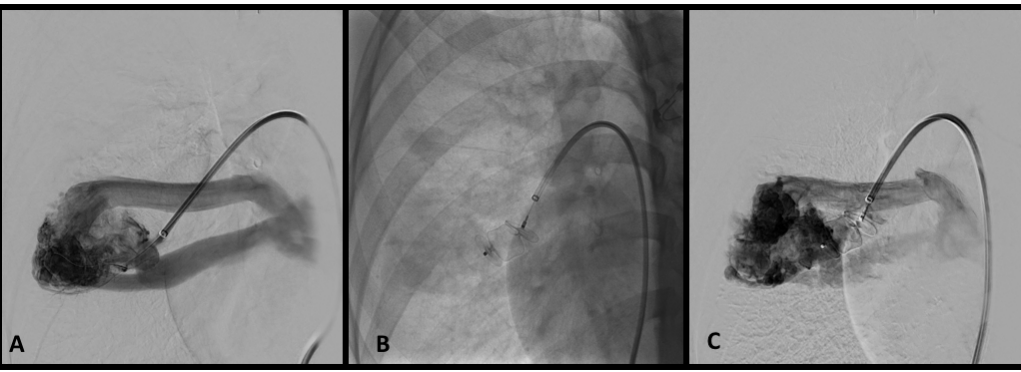
Treatment sequence of the voluminous arteriovenous malformation (case 2). (A) Selective catheterization of arteriovenous malformation in the lower right lobe; and (B) deployment of the Amplatzer^®^ plug. Control arteriography (C) still shows flow through the plug mesh, which is to be expected immediately after deployment.

**Figure 9:**
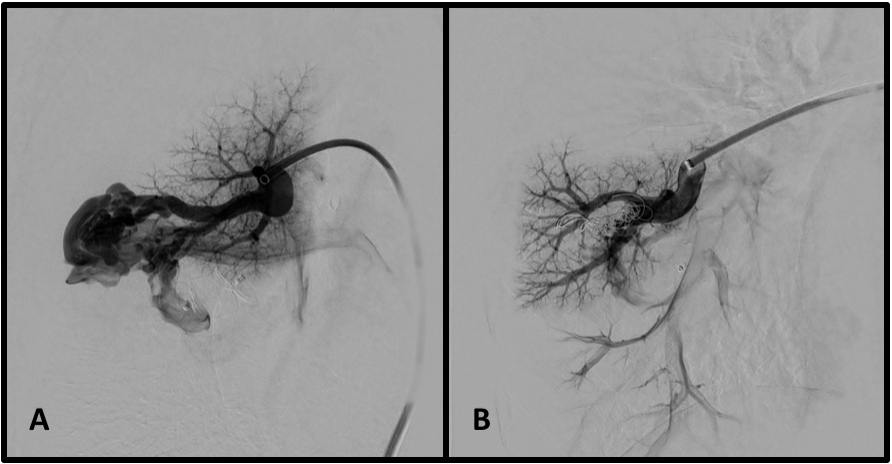
Embolization of arteriovenous malformations in case 2. Selective catheterization of arteriovenous malformation in the lower right lobe (A) and control angiography after embolization with four pushable coils (B).

**Figure 10:**
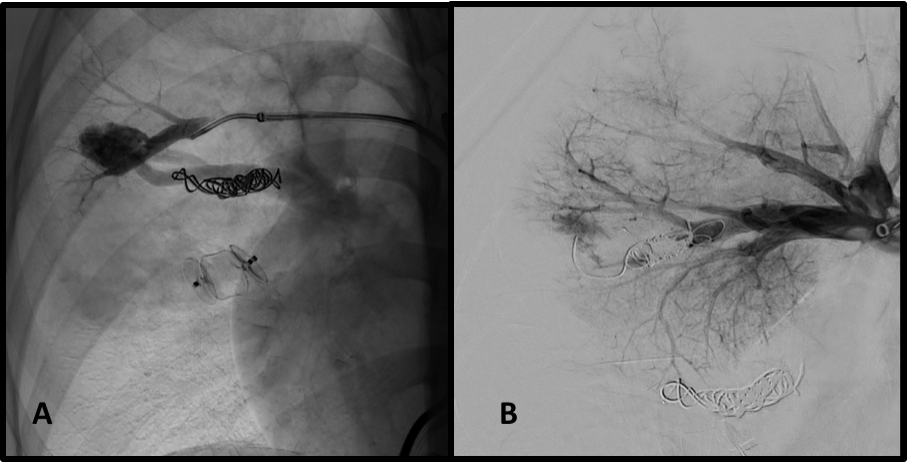
Embolization of arteriovenous malformations in case 2. Selective catheterization of the arteriovenous malformation in the upper right lobe (A) and control angiography after embolization with two pushable coils (B).

The patient underwent embolization of the AVMs using the same technique described in case 1. The voluminous AVM in the lower right lobe was treated with an Amplatzer II plug^®^ 16 mm (Figure 8). The smaller AVMs were embolized with pushable Nester^®^ 14-12 coils, four units in the lower lobe (Figure 9A and 9B) and two units in the upper lobe (Figure 10A and 10B). Postoperative recovery was free from complications and the patient was discharged from hospital on POD 3.

**Figure 11:**
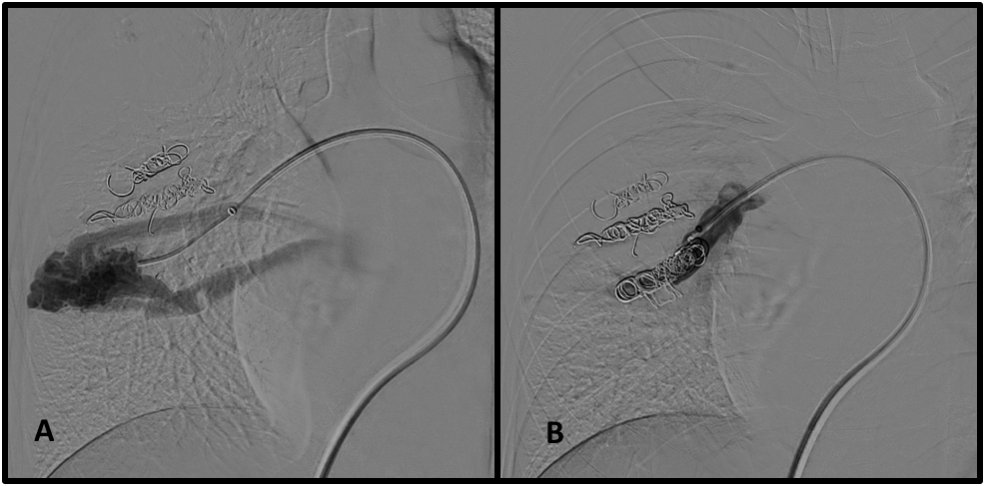
Reintervention in case 2. Selective catheterization and identification of a de novo arteriovenous malformation adjacent to the occluder plug deployed in the first procedure (A) and control angiography after embolization with ten pushable coils (B).

An angio-CT ordered for 12-month follow-up showed a large de novo AVM in the lower right lobe, adjacent to the embolization material. We decided to perform another embolization procedure, since this was a high-flow AVM involving a risk of higher-volume bleeding. We used 10 pushable Nester^®^ 14-12 coils (Figure 11A and 11B). The patient was discharged from hospital on POD 2. Four years after the first intervention, he remains in outpatient follow-up with the pulmonology service and is asymptomatic with preserved functional capacity.

## DISCUSSION

The ROWS syndrome is diagnosed on the basis of clinical criteria and can be confirmed by identification of two gene mutations (endoglin and ACVRL-1), which are present in 90% of mutations related to this disease.^4,5^

The signs and symptoms can be subtle, even in adulthood. Nosebleeding is the most common symptom, is present in more than 90% of the patients, is recurrent, and frequently causes anemia.^2,6^ Another characteristic lesion is macular telangiectasia, affecting the face, tongue, ears, hands, trunk, and feet.^7^

Pulmonary lesions include AVMs and, less frequently, pulmonary hypertension. The majority of the clinical complications of this disease are related to pulmonary AVMs causing L-R arteriovenous shunt.^8^

Up to 50% of patients with ROWS have pulmonary AVMs and there is generally a positive family history in these patients. In 30% of cases, AVMs are multiple, 10% are bilateral, and they are generally located in the lower lobes.^4,9,10^

The majority of patients are asymptomatic, even when they have AVMs causing L-R shunts. When these shunts exceed 25% of total blood volume, dyspnea, cyanosis, digital clubbing, and extracardiac murmurs can emerge. Hepatic AVMs can also be seen, in 32 to 78% of patients.^8^

The natural course of these lesions is not benign. Infections are related to L-R shunts and to emboli passing into the systemic circulation. Paradoxical emboli can cause cerebral abscess or infarction in 5 to 14% of patients and the risk may be higher in patients with multiple pulmonary AVMs, especially when the feeder artery exceeds 3 mm in diameter.^9^ Sites of infection and abscesses include kidneys, knees, spinal marrow, liver, and soft tissues.^8^ Overall mortality among patients with arteriovenous fistulas can be as high as 15.8%.^11^

ROWS is easily recognized in patients with the classic triad of nosebleeds, telangiectasia, and family history, but can be difficult to diagnose in many patients. In 2000, four diagnostic criteria, known as the Curaçao criteria, were defined to facilitate identification and diagnosis (Table 1).^6,12^

**Table 1:** Curaçao criteria.

**1.** Multiple telangiectasias at typical sites (face, lips, hands, and oral cavity)
**2.** Recurrent nosebleeds
**3.** AVMs with visceral involvement (pulmonary, hepatic, cerebral, gastrointestinal, or spinal)
**4.** Family history (first degree relative with ROWS)

Note: The diagnosis of ROWS is definitive if three criteria are present; possible or suspected if two criteria are positive; and improbable if only one criterion is identified. AVMs = arteriovenous malformations; ROWS = Rendu-Osler-Weber syndrome.

Up until the end of the 1970s, the treatment choices for pulmonary AVMs were pulmonary lobectomy, wedge resection of the segment involved, or direct surgical ligature of the arteriovenous fistula. Nowadays, embolization has become the standard treatment, given its lower impact in terms of the morbidity and mortality accrued by the intervention.^10^

Selection of pulmonary AVMs for embolization is based on the diameter of the feeder artery, although embolization may still be appropriate at diameters less than 3 mm. In several uncontrolled series, embolization has proven effective and demonstrated a good safety profile, with rare complications, high technical success rates, and significant improvement of oxygenation. Pulmonary AVMs can recanalize after successful treatment in up to 25% of cases.^13^ Small pulmonary AVMs may grow in up to 18% of cases. Recanalization of previously embolized feeder arteries is the most common mechanism leading to identification of persistent AVMs. Amplatzer^®^ plugs used alone or in conjunction with coils can reduce procedure time and reduce recanalization.^14,15^ The risk factors for recanalization after coil embolization include feeder artery enlargement, proximal placement of coils, and too few units of coils employed.^14^

The embolization procedure can provoke benign complications, such as pain and pleural effusion, which improve with symptomatic treatment. Significant complications such as symptomatic pulmonary infarct and systemic device migration through the AVM are observed rarely and can be avoided by careful placement and fitting of the sizes of the coils or occluder plugs to the vessels of the AVM. Gaseous emboli, transitory angina, cardiac arrhythmia, deep venous thrombosis, and pneumothorax are even rarer.^10^

Long-term follow-up is described using angio-CT, which identifies evolution of the AVM after treatment and growth of small residual AVMs, which are common in HHT.^6^

Pulmonary AVMs are associated with the more severe complications of ROWS. Endovascular treatment offers safety and control of complications and is currently the treatment of choice for these lesions.

This study was approved by the Research Ethics Committee at the institution to which the authors are affiliated (decision number 4.783.689, Ethics Appraisal Submission Certificate: 47568021.5.0000.5259).
