# Supplementary material for: Pulmonary arteriovenous malformations in Rendu-Osler-Weber syndrome
Source: J Vasc Bras. 2024 Apr 8;23:e20230133. doi: 10.1590/1677-5449.202301332 (PMC11042540; doi:10.1590/1677-5449.202301332)
Supplement: Translation

# Translation: Malformações arteriovenosas pulmonares na síndrome de Rendu-Osler-Weber

**DOI:** 10.1590/1677-5449.202301331

**Authors:** Cristiane Ferreira de Araújo-Gomes, Carlos Eduardo Virgini-Magalhães, Leonardo Silveira de Castro, Eduardo de Oliveira Rodrigues, Alex Antunes Bezerra, Monica Rochedo Mayall, Cristina Ribeiro Riguetti-Pinto, Felipe Borges Fagundes

**Affiliations:** 1 Universidade do Estado do Rio de Janeiro - UERJ, Hospital Universitário Pedro Ernesto - HUPE, Rio de Janeiro, RJ, Brasil.

**Keywords:** embolização terapêutica, fístula arteriovenosa, telangiectasia hemorrágica hereditária, Rendu-Osler-Weber

## Abstract

A síndrome de Rendu-Osler-Weber, também conhecida como telangiectasia hemorrágica hereditária, é uma doença hereditária autossômica dominante. Ela é caracterizada pela presença de múltiplas malformações arteriovenosas e telangiectasias. Este artigo relata dois casos de pacientes com síndrome de Rendu-Osler-Weber que apresentaram malformações arteriovenosas pulmonares e foram submetidos a tratamento endovascular com sucesso. Uma breve revisão da literatura mostra que até 50% dos pacientes com a síndrome têm malformações arteriovenosas pulmonares e geralmente há um histórico familiar positivo nesses pacientes. Em 30% dos casos, elas são múltiplas e estão associadas a complicações mais graves da doença. A maioria dos pacientes é assintomática, mesmo na presença de malformações arteriovenosas com *shunt* direito-esquerdo. Quando esses *shunts* excedem 25% do volume total de sangue, podem surgir dispneia, cianose, baqueteamento digital e sopros extracardíacos. O tratamento endovascular oferece segurança e controle das complicações da telangiectasia hemorrágica hereditária, sendo atualmente o tratamento de escolha para essas lesões.

## INTRODUÇÃO

A síndrome de Rendu-Osler-Weber (SROW), ou telangiectasia hemorrágica hereditária (THH), é uma doença autossômica dominante com prevalência estimada de 1/5.000^1^ . É caracterizada pela presença de múltiplas malformações arteriovenosas (MAVs) e telangiectasias. As MAVs podem se desenvolver ou crescer ao longo do tempo no fígado, pulmão e cérebro. Frequentemente formam grandes *shunts* direito-esquerdo (D-E), levando a aumento do débito cardíaco e dessaturação do sangue arterial e sendo a origem de êmbolos venosos que chegam à circulação periférica^2^ . A SROW encurta a expectativa de vida em média 7 anos^3^ .

Este trabalho descreve o tratamento endovascular em dois casos de pacientes com SROW que apresentavam MAVs pulmonares e faz uma breve revisão da literatura sobre essa doença rara.

## PARTE IA - SITUAÇÃO CLÍNICA

Uma paciente de 47 anos, do sexo feminino, iniciou um quadro clínico em 2018 com dispneia progressiva e queda da saturação de oxigênio (SaO_2_). A investigação clínica revelou ausculta pulmonar normal, dispneia aos médios esforços e baqueteamento digital. Na sua história patológica pregressa, destacavam-se acidente vascular encefálico isquêmico aos 26 anos de idade, sem sequelas motoras ou funcionais, internação por pneumonia com necessidade de ventilação mecânica e cirurgia prévia devido a abscessos renal e hepático há 11 meses. Relatava ainda história de tratamento prévio para eritrocitose, leucopenia, trombocitopenia e poliglobulia.

A angiotomografia (angio-TC) de tórax confirmou a imagem de MAVs (Figura 1) com espirometria normal. A deterioração do quadro respiratório com dispneia aos mínimos esforços e a SaO_2_ menor que 65% em ar ambiente impuseram o dilema entre o tratamento conservador dependente de oxigenoterapia domiciliar e a intervenção. Entre as opções de intervenção, a embolização das MAVs era a melhor alternativa, já que o tratamento cirúrgico convencional apresentava maior risco e morbidade para o caso clínico em questão.

**Figura 1:** Angiotomografia de tórax (caso 1) com lesões nodulares hipervascularizadas características das malformações arteriovenosas.

## PARTE IIA - O QUE FOI FEITO

Foi solicitada a internação para tratamento endovascular das MAVs pulmonares. O procedimento foi realizado sob anestesia geral, com a paciente mantendo SaO_2_ em torno de 80% durante todo ato cirúrgico, a despeito da oxigenoterapia instituída.

Realizamos um acesso femoral pela técnica de Seldinger e o cateterismo das artérias pulmonares com identificação das MAVs (Figura 2). Foram feitos o cateterismo seletivo de cada MAV e a embolização de acordo com os seus diâmetros. A MAV do lobo inferior esquerdo foi embolizada utilizando-se seis molas de destaque livre Nester^®^ 14-12 (Figura 3A e 3B). A MAV de menor calibre, periférica, foi embolizada apenas com uma mola de destaque livre Nester^®^ 14-12 (Figura 4A e 4B).

**Figura 2:** Arteriografia seletiva identificando volumosas malformações arteriovenosas pulmonares (caso 1).

**Figura 3:** Embolização de malformações arteriovenosas do caso 1. (A) Cateterismo seletivo da malformação arteriovenosa em lobo inferior direito; e (B) resultado imediato após embolização com seis molas de destaque livre.

**Figura 4:** Embolização de malformações arteriovenosas do caso 1. (A) Cateterismo seletivo de malformação arteriovenosa periférica; e (B) liberação de mola de destaque livre.

Outras MAVs calibrosas (Figura 5A) foram embolizadas junto com um plugue oclusor Amplatzer II^®^ de 16 mm. Utilizamos o último anel do plugue para ocluir o óstio da artéria nutridora da MAV menor (Figura 5B, seta). Imediatamente após a liberação dos dispositivos, houve aumento da SaO_2_ para 96%.

**Figura 5:** Embolização de malformações arteriovenosas do caso 1. (A) Cateterismo seletivo de malformação arteriovenosa volumosa; e (B) liberação de plug Amplatzer^®^. Detalhe (seta) da liberação do último anel do plug para ocluir a malformação arteriovenosa de menor calibre.

No segundo dia de pós-operatório (DPO), a paciente apresentou dor torácica intensa, tendo sido tratada com analgesia, e houve resolução completa do quadro após 48 horas. Ela recebeu alta hospitalar no quinto DPO, mantendo a SaO_2_ em 96%. Após 4 anos da intervenção, a paciente segue em acompanhamento ambulatorial com a Pneumologia, apresentando estabilidade clínica e capacidade funcional preservada.

## PARTE IB - SITUAÇÃO CLÍNICA

Um paciente assintomático de 20 anos, do sexo masculino, foi encaminhado para uma investigação clínica devido ao achado radiológico de massa pulmonar em hemitórax direito em exame admissional. Na anamnese dirigida, relatou episódios autolimitados de epistaxe durante a infância e adolescência e sangramentos na gengiva e na língua, sem repercussões. A história familiar era positiva para episódios semelhantes de epistaxe. No exame físico, a única alteração encontrada foi a presença de pequena telangiectasia na cavidade oral (Figura 6).

**Figura 6:** Exame físico do paciente (caso 2) normal, exceto pela presença de pequena telangiectasia na cavidade oral.

O ecocardiograma transtorácico pela técnica de microbolhas revelou fração de ejeção de 69%, com *shunt* pulmonar D-E. A endoscopia digestiva alta evidenciou angiectasias vasculares gástricas e varizes de esôfago de pequeno calibre (BAVENO VI). A angio-TC de tórax, além de confirmar a MAV nos lobos superior e inferior do pulmão direito, identificou múltiplas imagens nodulares hipervascularizadas no fígado, também compatíveis com MAVs (Figura 7).

**Figura 7:** A angiotomografia identificou múltiplas imagens nodulares hipervascularizadas no parênquima hepático compatíveis com malformações arteriovenosas (caso 2).

Nesse caso, o dilema que se apresentava, em um paciente assintomático, era optar entre a conduta conservadora e a intervenção das lesões de forma profilática. Devido ao risco de grande sangramento da lesão e por se tratar de um paciente jovem, indicamos o tratamento endovascular das lesões pulmonares.

## PARTE IIB - O QUE FOI FEITO

O paciente foi submetido à embolização das MAVs com a mesma técnica descrita no caso 1. Na MAV volumosa do lobo inferior direito, foi utilizado um plugue Amplatzer II^®^ de 16 mm (Figura 8). Nas MAVs menores, foram utilizadas molas de destaque livre Nester^®^ 14-12, quatro unidades no lobo inferior (Figura 9A e 9B) e duas unidades no lobo superior (Figura 10A e 10B). O pós-operatório evoluiu sem intercorrências, e o paciente teve alta hospitalar no terceiro DPO.

**Figura 8:** Sequência do tratamento de malformação arteriovenosa volumosa (caso 2). (A) Cateterismo seletivo de malformação arteriovenosa no lobo inferior direito; e (B) liberação do plug Amplatzer^®^. A arteriografia de controle (C) ainda demonstra fluxo através da malha do plugue, o que é esperado imediatamente após a sua liberação.

**Figura 9:** Embolização de malformações arteriovenosas do caso 2. Cateterismo seletivo de malformação arteriovenosa do lobo inferior direito (A) e controle angiográfico após embolização de quatro molas de destaque livre (B).

**Figura 10:** Embolização de malformações arteriovenosas do caso 2. Cateterismo seletivo de malformação arteriovenosa do lobo superior direito (A) e controle angiográfico após embolização de duas molas de destaque livre (B).

Na angio-TC de seguimento de 12 meses, observamos nova MAV volumosa no lobo inferior direito, adjacente ao material de embolização. Optamos pela realização de nova embolização, por se tratar de MAV de alto fluxo com risco de sangramento de maior volume. Utilizamos 10 molas de destaque livre Nester^®^ 14-12 (Figura 11A e 11B). O paciente recebeu alta hospitalar no segundo DPO. Após 4 anos da primeira intervenção, ele segue em acompanhamento ambulatorial com a Pneumologia, assintomático e com capacidade funcional preservada.

**Figura 11:** Reintervenção no caso 2. Cateterismo seletivo e identificação de nova malformação arteriovenosa adjacente ao plugue oclusor da primeira cirurgia (A) e controle angiográfico após nova embolização com dez molas de destaque livre (B).

## DISCUSSÃO

A síndrome SROW tem o seu diagnóstico estabelecido por critérios clínicos, que pode ser confirmado pela identificação de mutações de dois genes (endoglina e ACVRL-1), presentes em 90% das mutações relacionadas à doença^4,5^ .

Os sinais e sintomas podem ser sutis mesmo na idade adulta. A epistaxe é o sintoma mais comum, estando presente em mais de 90% dos pacientes, é recorrente e frequentemente leva à anemia^2,6^ . Outra lesão característica é a telangiectasia macular, que ocorre na face, língua, orelhas, mãos, tronco e pés^7^ .

As lesões pulmonares incluem as MAVs e, menos frequentemente, a hipertensão pulmonar. A maioria das complicações clínicas da doença está relacionada a MAVs pulmonares com *shunt* arteriovenoso D-E^8^ .

Até 50% dos pacientes com SROW têm MAVs pulmonares, e geralmente há histórico familiar positivo nesses pacientes. Em 30% dos casos, são múltiplas, 10% são bilaterais e estão geralmente localizadas nos lobos inferiores^4,9,10^ .

A maioria dos pacientes é assintomática, mesmo em presença de MAVs com *shunts* D-E. Quando esses *shunts* excedem 25% do volume total de sangue, podem surgir dispneia, cianose, baqueteamento digital e sopros extracardíacos. As MAVs hepáticas também podem ser encontradas em 32 a 78% dos pacientes^8^ .

O curso natural da lesão não é benigno. As infecções estão relacionadas ao *shunt* D-E e à passagem de êmbolos para a circulação sistêmica. Os êmbolos paradoxais podem resultar em infarto ou abscesso cerebral em 5 a 14% dos pacientes, e o risco pode ser maior em pacientes com múltiplas MAVs pulmonares, especialmente quando a artéria nutridora excede 3 mm de diâmetro^9^ . Os sítios de infecção e abscessos incluem rim, joelho, medula espinhal, fígado e tecidos moles^8^ . A mortalidade geral em pacientes que possuem fístula arteriovenosa pode chegar a 15,8%^11^ .

A SROW é facilmente reconhecida em indivíduos que apresentam a tríade clássica de epistaxe, telangiectasia e história familiar, mas é de difícil diagnóstico em muitos pacientes. No ano 2000, foram definidos quatro critérios diagnósticos, conhecidos como critérios de Curaçao, para facilitar sua identificação e diagnóstico (Tabela 1)^6,12^ .

**Tabela 1:** Critérios de Curaçao.

**1.** Telangiectasias múltiplas em locais típicos (face, lábios, mãos e cavidade oral)
**2.** Epistaxes recorrentes
**3.** MAVs com comprometimento visceral (pulmonares, hepáticas, cerebrais, gastrointestinais ou medulares)
**4.** História familiar (parente de primeiro grau com SROW)

Nota: O diagnóstico da SROW é definitivo se três critérios estiverem presentes; possível ou suspeito quando dois critérios forem positivos; e improvável se apenas um critério for identificado. MAVs = malformações arteriovenosas; SROW = síndrome de Rendu-Osler-Weber.

Até o final da década de 1970, os tratamentos de escolha para as MAVs pulmonares eram lobectomia pulmonar, ressecção em cunha do segmento acometido ou ligadura cirúrgica direta da fístula arteriovenosa. Atualmente, a embolização tornou-se a terapia padrão, dado o menor impacto na morbimortalidade agregado pela intervenção^10^ .

A seleção das MAVs pulmonares para embolização é baseada no diâmetro da artéria nutridora, embora a embolização com diâmetros menores que 3 mm possa ser apropriada. A embolização demonstrou, em várias séries não controladas, ser eficaz e ter um bom perfil de segurança, com raras complicações, altas taxas de sucesso técnico e significativa melhora da oxigenação. Após um tratamento bem-sucedido, as MAVs pulmonares podem recanalizar em até 25% dos casos^13^ . Pode ocorrer crescimento de pequenas MAVs pulmonares em até 18% dos casos. A recanalização de artérias nutridoras previamente embolizadas é o mecanismo mais comum que leva à identificação de MAVs persistentes. Os plugues Amplatzer^®^, utilizados sozinhos ou em conjunto com molas, podem reduzir o tempo do procedimento e a recanalização^14,15^ . Os fatores de risco para recanalização após a embolização com mola incluem aumento da artéria nutridora, colocação da mola proximal e poucas unidades de mola utilizadas^14^ .

O procedimento de embolização pode apresentar complicações benignas, como dor e derrame pleural, que melhoram com o tratamento sintomático. Complicações importantes, como infarto pulmonar sintomático e migração sistêmica do dispositivo através da MAV, raramente são encontradas e podem ser evitadas pelo posicionamento cuidadoso e pela adaptação do tamanho das molas ou plugues oclusores aos vasos da MAV. Embolia gasosa, angina transitória, arritmia cardíaca, trombose venosa profunda e pneumotórax são ainda mais raros^10^ .

O acompanhamento no longo prazo é descrito usando a angio-TC, que identifica a evolução da MAV após o tratamento e o crescimento de pequenas MAVs residuais, comuns na THH^6^ .

As MAVs pulmonares estão associadas às complicações mais graves da SROW. O tratamento endovascular oferece segurança e controle das complicações, sendo atualmente o tratamento de escolha para essas lesões.

Este estudo foi aprovado pelo Comitê de Ética em Pesquisa a que se vinculam os autores (parecer 4.783.689, Certificado de Apresentação de Apreciação Ética: 47568021.5.0000.5259).
